# Binding of Sudan II and IV to lecithin liposomes and *E. coli *membranes: insights into the toxicity of hydrophobic azo dyes

**DOI:** 10.1186/1472-6807-7-16

**Published:** 2007-03-27

**Authors:** Lu Li, Hong-Wen Gao, Jiao-Rong Ren, Ling Chen, Yu-Cheng Li, Jian-Fu Zhao, He-Ping Zhao, Yuan Yuan

**Affiliations:** 1 State Key Laboratory of Pollution Control and Resource Reuse, College of Environmental Science and Engineering, Tongji University, Shanghai 200092, P. R. China; 2 Key Laboratory of Yangtze Water Environment of Ministry of Education, College of Environmental Science and Engineering, Tongji University, Shanghai 200092, P. R. China; 3 School of Life Science, Anhui University, Hefei 230039, P. R. China

## Abstract

**Background:**

Sudan red compounds are hydrophobic azo dyes, still used as food additives in some countries. However, they have been shown to be unsafe, causing tumors in the liver and urinary bladder in rats. They have been classified as category 3 human carcinogens by the International Agency for Research on Cancer. A number of hypotheses that could explain the mechanism of carcinogenesis have been proposed for dyes similar to the Sudan red compounds. Traditionally, investigations of the membrane toxicity of organic substances have focused on hydrocarbons, e.g. polycyclic aromatic hydrocarbons (PAHs), and DDT. In contrast to hydrocarbons, Sudan red compounds contain azo and hydroxy groups, which can form hydrogen bonds with the polar head groups of membrane phospholipids. Thus, entry may be impeded. They could have different toxicities from other lipophilic hydrocarbons. The available data show that because these compounds are lipophilic, interactions with hydrophobic parts of the cell are important for their toxicity. Lipophilic compounds accumulate in the membrane, causing expansion of the membrane surface area, inhibition of primary ion pumps and increased proton permeability.

**Results:**

This work investigated the interactions of the amphiphilic compounds Sudan II and IV with lecithin liposomes and live *Escherichia coli (E. coli)*. Sudan II and IV binding to lecithin liposomes and live *E. coli *corresponds to the Langmuir adsorption isotherm. In the Sudan red compounds – lecithin liposome solutions, the binding ratio of Sudan II to lecithin is 1/31 and that of Sudan IV to 1/314. The binding constant of the Sudan II-lecithin complex is 1.75 × 10^4 ^and that of the Sudan IV-lecithin complex 2.92 × 10^5^. Besides, the influences of pH, electrolyte and temperature were investigated and analyzed quantitatively. In the Sudan red compounds – *E.coli *mixture, the binding ratios of Sudan II and Sudan IV to *E.coli *membrane phospholipid are 1/29 and 1/114. The binding constants of the Sudan II – and Sudan IV- *E.coli *membrane phospholipid complexes are 1.86 × 10^4 ^and 6.02 × 10^4^. Over 60% of Sudan II and 75% of Sudan IV penetrated into *E.coli*, in which 90% of them remained in the *E.coli *membrane.

**Conclusion:**

Experiments of Sudan II and IV binding to lecithin liposomes and live *E. coli *indicates that amphiphilic compounds may besequestered in thelecithin liposomes and membrane phospholipid bilayer according to the Langmuir adsorption law. Penetration into the cytosol was impeded and inhibited for Sudan red compounds. It is possible for such compounds themselves (excluding their metabolites and by-products)not result directly in terminal toxicity. Therefore, membrane toxicity could be manifested as membrane blocking and membrane expansion. The method established here may be useful for evaluating the interaction of toxins with membranes.

## Background

Many toxic chemicals, *e.g*. drugs and harmful additives that are formed or utilized in daily life, accumulate persistently in biological systems. Sudan red compounds I – IV, which are hydrophobic azo dyes, are still used as food additives in some countries because of their low cost and bright color [1] but they have been shown to be unsafe, causing tumors in the liver or urinary bladder [2] in rats. Sudan I – IV have been classified as category 3 carcinogens to humans by International Agency for Research on Cancer [3], and the European Union does not allow Sudan dyes as food additives [4,5]. A number of hypotheses that could explain the mechanism of carcinogenesis have been proposed for dyes similar to the Sudan red compounds [6].

Traditionally, investigations of lipid peroxidation damage by Sudan red compounds have been performed only on mouse and rabbit sera. Recently, Stiborova [7] compared experimental animals with humans and determined that cytochromes P450 and live microsomes are essential for extrapolating animal carcinogenicity data to human health risk assessment. Although Sudan dyes are indirect carcinogens, they generate metabolites that are converted to several active mutagens and carcinogens in humans [8,9], such as aniline or 1-amido-2-naphthol, which can be metabolized by hepatic microsomes into benzene and naphthol. These end products can then combine with DNA and RNA to destroy cells [10].

To cause terminal toxicity, a chemical must penetrate the cell so as to affect the function of a target biomolecule. However, the cell membrane, which consists of a lipid bilayer and membrane proteins along with sugar polymers as a support layer, acts as a natural barrier and often plays a protective role in normal cellular activity. Cell membranes perform a number of essential functions such as transport of nutrients, ion conduction, signal transduction, cell migration, etc. The plasma membrane represents the primary permeability barrier of a cell and therefore regulates the inflow of substrates and the outflow of products. Normal cell activity will be seriously compromised if the cell is persistently exposed to a toxic medium. The partitioning of hydrocarbons in membrane buffer systems has been studied [11,12]. The available data show that because of their lipophilic character, these compounds interact with hydrophobic parts of the cell, and this is important in the mechanism of toxicity. Lipophilic compounds accumulate in the membrane, causing "expansion" of the membrane surface area, inhibition of primary ion pumps and increased proton permeability [12].

The interaction of lipophilic compounds with the phospholipid bilayer causes dramatic changes in cell membrane structure. For example, accumulation of such compounds in the hydrophobic part of the membrane will disturb the interactions between the phospholipid acyl chains, modifying membrane fluidity and eventually leading to swelling of the bilayer. Furthermore, the lipid annuli that surround membrane-embedded proteins will also change, possibly altering protein conformations [13]. Because Sudan red compounds have groups capable of forming hydrogen bonds, their toxic effects on the membrane should differ from those of apolar hydrocarbons.

In this study, we have investigated the interactions of the amphiphilic compounds Sudan II and IV with both lecithin liposomes and live *E. coli *membrane phospholipids. Polar compounds with hydrogen-bond forming groups *e.g*. -OH, = O, -NH_2_, -N = N- have been shown to accumulate on the external surfaces of membranes according to the Langmuir adsorption isotherm [14]. Entry into the liposome center and cytosol is impeded for amphiphilic compounds, so it is possible for them not to cause the terminal toxicity. However, their metabolites and by-products could be terminally toxic, possibly formed by exo-enzyme or photocatalysis [15-17]. Membrane toxicity could therefore be manifested as membrane blocking and membrane expansion. The method established in the present work could be valuable for evaluating the interaction of toxins with membranes and will be applicable to research in the environmental and life sciences, and medicine.

## Results and discussion

### Absorption spectra and structural analysis

**Figure 1:**
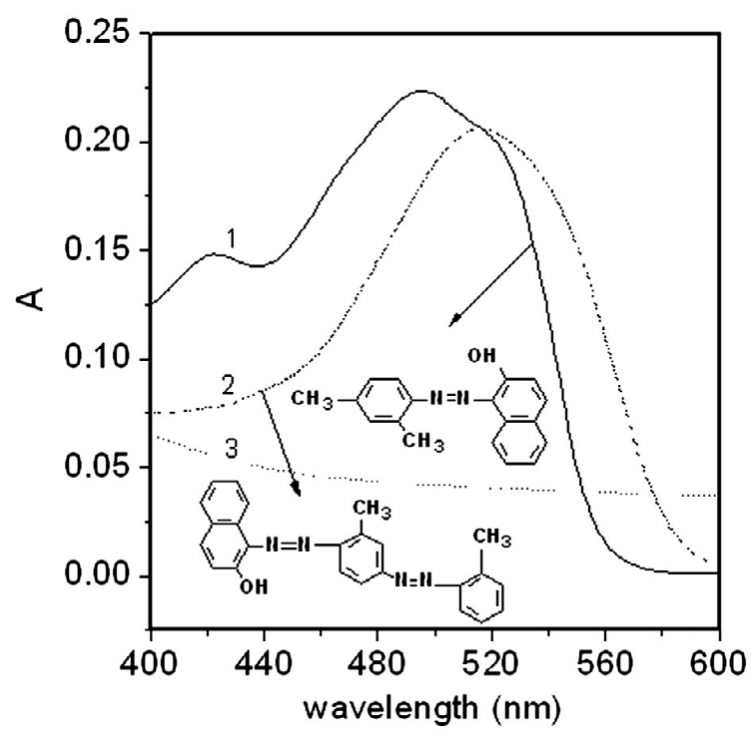
**Visible spectra of solutions containing**: 1, Sudan II (14 μM) in ethanol; 2, Sudan IV (6.3 μM) in ethanol; 3, lecithin liposomes (10 mg/ml).

Because Sudan II and IV are amphiphilic compounds, their absorption spectra were measured in ethanol solution (Figure 1) and showed wavelength maxima of 495 nm for Sudan II and 515 nm for Sudan IV. Both compounds contain the polar groups -OH and -N = N-, which may form hydrogen bonds with the other polar groups. A methyl group of Sudan II is replaced by a methylphenylazo group in Sudan IV, extending the conjugation of double bonds, so that the *λ*_max _of Sudan IV is increased. Since Sudan IV has one more lipophilic phenyl ring than Sudan II, Sudan IV is significantly less hydrophilic.

In order to investigate the interaction of these compounds with lecithin liposomes, the mixtures must be centrifuged. However, the centrifugal pellet appears light yellow in ethanol solution, so the background interference was estimated by a dual-wavelength correction [18] as follows:

Aλ2S/L=a(Aλ2−bAλ1)a−b     (1)
 MathType@MTEF@5@5@+=feaafiart1ev1aaatCvAUfKttLearuWrP9MDH5MBPbIqV92AaeXatLxBI9gBaebbnrfifHhDYfgasaacH8akY=wiFfYdH8Gipec8Eeeu0xXdbba9frFj0=OqFfea0dXdd9vqai=hGuQ8kuc9pgc9s8qqaq=dirpe0xb9q8qiLsFr0=vr0=vr0dc8meaabaqaciaacaGaaeqabaqabeGadaaakeaacqWGbbqqdaqhaaWcbaaccaGae8hiaaIae8hiaaIae8hiaaIae8hiaaIae8hiaaIae8hiaaIae8hiaaIae83UdWMaeGOmaidabaGaem4uamLaei4la8IaemitaWeaaOGaeyypa0ZaaSaaaeaacqWGHbqycqGGOaakcqWGbbqqdaWgaaWcbaGaeq4UdWMaeGOmaidabeaakiabgkHiTiabdkgaIjabdgeabnaaBaaaleaacqaH7oaBcqaIXaqmaeqaaOGaeiykaKcabaGaemyyaeMaeyOeI0IaemOyaigaaiaaxMaacaWLjaWaaeWaaeaacqaIXaqmaiaawIcacaGLPaaaaaa@4E8D@

where

a=Aλ2SAλ1S
 MathType@MTEF@5@5@+=feaafiart1ev1aaatCvAUfKttLearuWrP9MDH5MBPbIqV92AaeXatLxBI9gBaebbnrfifHhDYfgasaacH8akY=wiFfYdH8Gipec8Eeeu0xXdbba9frFj0=OqFfea0dXdd9vqai=hGuQ8kuc9pgc9s8qqaq=dirpe0xb9q8qiLsFr0=vr0=vr0dc8meaabaqaciaacaGaaeqabaqabeGadaaakeaacqWGHbqycqGH9aqpdaWcaaqaaiabdgeabnaaDaaaleaacqaH7oaBcqaIYaGmaeaacqWGtbWuaaaakeaacqWGbbqqdaqhaaWcbaGaeq4UdWMaeGymaedabaGaem4uamfaaaaaaaa@392F@ and b=Aλ2LAλ1L
 MathType@MTEF@5@5@+=feaafiart1ev1aaatCvAUfKttLearuWrP9MDH5MBPbIqV92AaeXatLxBI9gBaebbnrfifHhDYfgasaacH8akY=wiFfYdH8Gipec8Eeeu0xXdbba9frFj0=OqFfea0dXdd9vqai=hGuQ8kuc9pgc9s8qqaq=dirpe0xb9q8qiLsFr0=vr0=vr0dc8meaabaqaciaacaGaaeqabaqabeGadaaakeaacqWGIbGycqGH9aqpdaWcaaqaaiabdgeabnaaDaaaleaacqaH7oaBcqaIYaGmaeaacqWGmbataaaakeaacqWGbbqqdaqhaaWcbaGaeq4UdWMaeGymaedabaGaemitaWeaaaaaaaa@3915@

and *λ*_1 _and *λ*_2 _are the measurement wavelengths. *λ*_1 _was selected at 400 nm and *λ*_2 _at 495 nm for Sudan II and 515 nm for Sudan IV. The symbol *A*^S/L^_*λ*2 _is the real absorbance of a Sudan compound adsorbed by the lecithin liposomes; *A*_*λ*1 _and *A*_*λ*2 _are the absorbances of the above solutions at *λ*_1 _and *λ*_2_. Both *a *and *b *are correction constants. *A*^*L*^_*λ*1 _and *A*^*L*^_*λ*2 _are the absorbances of a lecithin-ethanol solution at *λ*_1 _and *λ*_2 _without any Sudan compound and *A*^*s*^_*λ*1 _and *A*^*S*^_*λ*2 _are those of a Sudan compound in ethanol solution without liposomes. For Sudan II, *a *= 1.66 and *b *= 0.642; for Sudan IV, *a *= 2.73 and *b *= 0.621. These values were calculated from curves 1 – 3 in Figure 1.

### Interaction of liposomes with the Sudan red compounds

**Figure 2:**
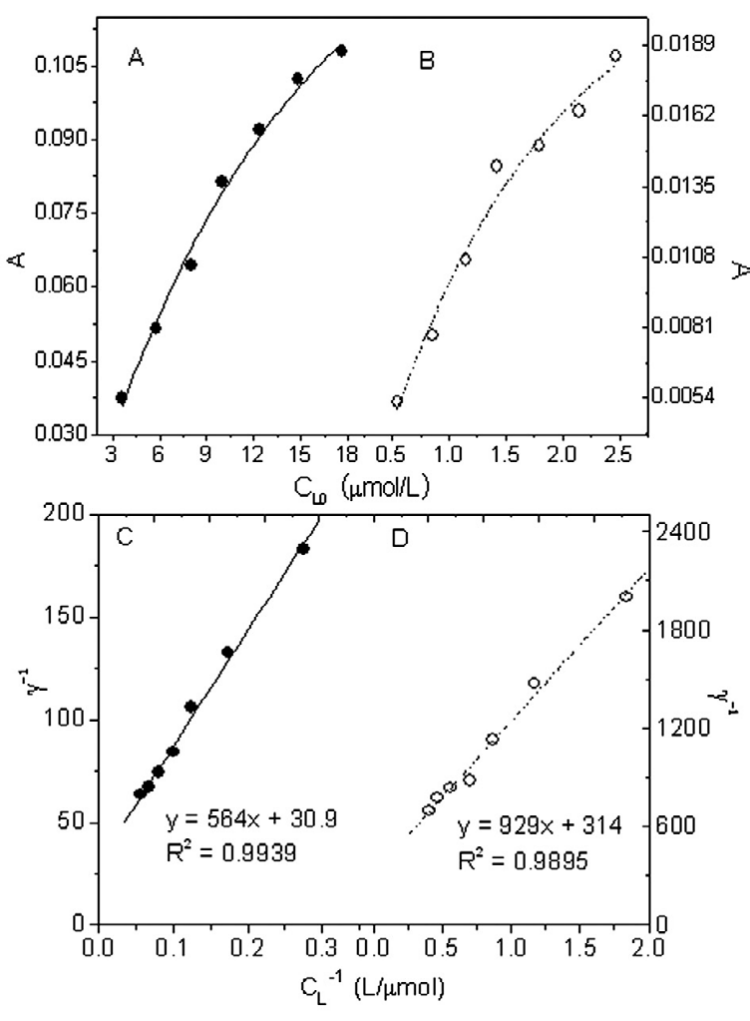
**Absorbance measurements of the solutions containing 0.20 mg/ml lecithin liposomes and Sudan II and IV and their linear regression**. A: Sudan II in lecithin liposomes was dissolved in ethanol and the absorbance was measured at 495 nm; B: Sudan IV was dissolved in ethanol and the absorbance was measured at 515 nm; C: plots of *γ*^-1 ^vs. C_L_^-1 ^for the interaction of Sudan II with liposomes; D: as C, but using Sudan IV in place of Sudan II.

The absorptions of Sudan II and IV accumulated in liposomes are shown in Figure 2(A & B). Their free concentrations in aqueous solution (*C*_L_) were calculated from Eqs.2 and 4 in Table 1. We attempted to analyze the interaction of liposomes with Sudan red compounds according to the Langmuir isothermal equation [14]:

1γ=1N+1KNCL     (6)
 MathType@MTEF@5@5@+=feaafiart1ev1aaatCvAUfKttLearuWrP9MDH5MBPbIqV92AaeXatLxBI9gBaebbnrfifHhDYfgasaacH8akY=wiFfYdH8Gipec8Eeeu0xXdbba9frFj0=OqFfea0dXdd9vqai=hGuQ8kuc9pgc9s8qqaq=dirpe0xb9q8qiLsFr0=vr0=vr0dc8meaabaqaciaacaGaaeqabaqabeGadaaakeaadaWcaaqaaiabigdaXaqaaGGaciab=n7aNbaacqGH9aqpdaWcaaqaaiabigdaXaqaaiabd6eaobaacqGHRaWkdaWcaaqaaiabigdaXaqaaiabdUealjabd6eaojabdoeadnaaBaaaleaacqWGmbataeqaaaaakiaaxMaacaWLjaWaaeWaaeaacqaI2aGnaiaawIcacaGLPaaaaaa@3CD8@

**Figure 3:**
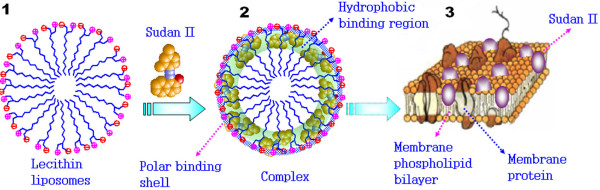
**Cartoon illustrating the proposed Sudan red-liposome binding**. 1, Liposome sphere; 2, Sudan II – liposome binding pattern involving hydrogen bond and hydrophobic bond; 3, Sudan II – cell membrane binding pattern.

The symbol *γ *denotes the binding number of L in liposomes and *N *is the maximal binding constant. *K *is the stability constant of the binding product. The higher the value of *K *is and the stronger the binding becomes. Regression plots for *γ*^-1 ^vs. *C*_L_^-1 ^in the Sudan red compound-lecithin liposome solutions (Figure 2(C and D)) indicated an interaction that obeyed the classical adsorption isotherm, with *N*_Sudan-II _= 1/31 and *K*_Sudan-II _= 1.75 × 0^4 ^for the Sudan II-liposome system and *N*_Sudan-IV _= 1/314 and *K*_Sudan-IV _= 2.92 × 10^5 ^for the Sudan IV-liposome system. Thus, 31 of lecithin molecules bound with one of Sudan II, but 314 of lecithin molecules with one of Sudan IV. This 10-fold difference is attributed to the fact that the Sudan IV molecule is larger and has a greater steric effect than Sudan II. In addition, the Sudan IV-liposome complex is more stable than the Sudan II-liposome complex, as indicated by their *K *values. The possible reason is the greater number of polar and lipophilic groups (potential binding sites) in Sudan IV can form more hydrogen bonds with the lecithin headgroups as illustrated in Figure 3(1) and more lipophilic bonds with aliphatic group of lecithin. Sudan II binding to a liposome is sketched in Figure 3(2). The hydrogen bonds appear around the surface of the liposome to form a polar binding sphere. In contrast, Sudan II will tend to move towards the central hydrophobic core because its aryl groups interact hydrophobically with the lecithin. Because of the polar attraction of hydrogen bonds, Sudan II is difficult to insert into the lecithin liposome center. Therefore, Sudan II could be adsorbed around the inner surface of the lecithin liposome as shown in Figure 3(2).

The phospholipid micelle will bind a polar hydrophobic organic compound via polar bonds, e.g. ion pairing, hydrogen bonds and dispersion forces, corresponding to the molecular monolayer adsorption model. The units of various non-covalent bonds determine the binding type, binding position and interaction strength of an organic compound [19]. With respect to biological activity, non-covalent interactions are common because they are non-specific. A more complicated molecule with more active polar groups, *e.g*. Sudan IV, often forms a more stable complex. Thus, Sudan IV binding to lecithin liposomes cannot be reversed easily. The binding of Sudan II and IV by liposomes indicates thatentry into the liposome center may be impeded for them, as further argued on the basis of the following experiments.

### Effect of ionic strength, pH and temperature

**Figure 4:**
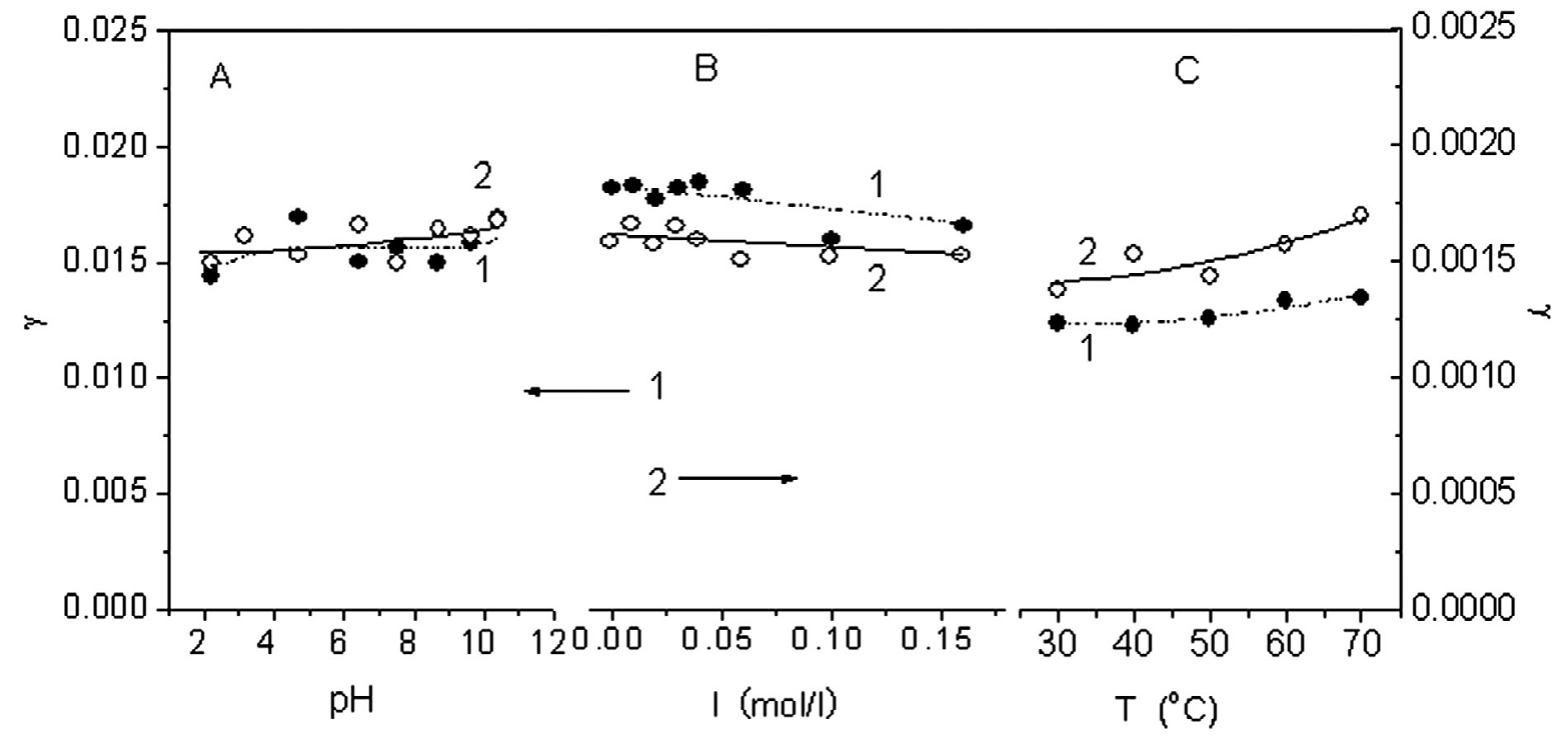
**Effects of pH (A), electrolyte (B) and temperature (C) on the interactions of Sudan red compounds with lecithin liposomes (0.20 mg/l)**. 1: 8 μM Sudan II and 2: 1 μM Sudan IV.

The effects of solution pH, electrolyte content and temperature on the interactions of Sudan red compounds with lecithin liposomes are shown in Figure 4. Curves A-1 and A-2 show that *γ *increases slowly when the pH increases from 2.18 to 10.38. If lecithin behaves similarly to an ionic surfactant [20], increasing pH favors the aggregation of organic substances. In addition, the hydroxyl groups of Sudan red compounds will form -O^-^anions in an alkaline media and will therefore bind firmly to the liposome surface by electrostatic interaction with the cationic lecithin headgroups. From curves B-1 and B-2, *γ *decreases with increasing ionic strength. Again comparing lecithin with an ionic surfactant, increasing ionic strength is often unfavorable for the aggregation of soluble organic substances [20]. The aggregation number of lecithin in a liposome micelle increase seriously with an increase of electrolyte concentration [21]. The number of liposomes suspending in solution will decrease then the total spherical surface of micelles area decreases. One Sudan red molecule will cover more lecithin molecules though increasing ionic strength may be favorable for hydrophobic Sudan red compounds to aggregate on the liposome. Curves C-1 and C-2 show that *γ *increases with increasing temperature. This is because the mean intermolecular distance in the liposome micelle increases at higher temperatures. Thus, the surface area of the lecithin liposome will increase, resulting in the binding of more Sudan red compounds.

### Analysis of *E. coli *growth state

**Figure 5:**
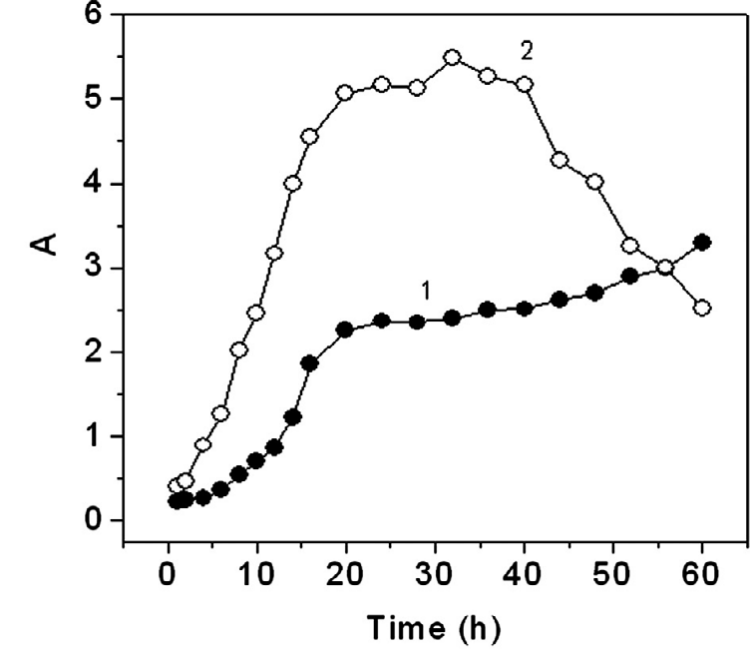
**Growth curve of *E. coli *at 37°C**. 1: turbidity of culture fluid at 600 nm at 48 h culture time; 2: variation of *E. coli *activity indicated by the absorbance measured at 555 nm with MTT.

In order to select *E. coli *in stable growth, the number of viable cells was measured at 555 nm by methylthiazolyldiphenyltetrazolium (MTT) assay [22] as shown in Figure 5. From curve 1, the number of *E. coli *cells increases exponentially during the first 20 h and then remains constant between 20 and 36 h. After 36 h, cell death occurs. From curve 2, the absorption of the culture medium at 600 nm remains almost constant between 20 and 36 h and then increases after 36 h. Curves 1 and 2 demonstrate not only that almost all the *E. coli *cells are alive but also that few cells were proliferating during the 20–36 h interval. Therefore, the cells were sampled at around 20 h for experiments to investigate the interactions between living cells and the Sudan red compounds.

### Distribution of Sudan red compounds in *E. coli*

**Figure 6:**
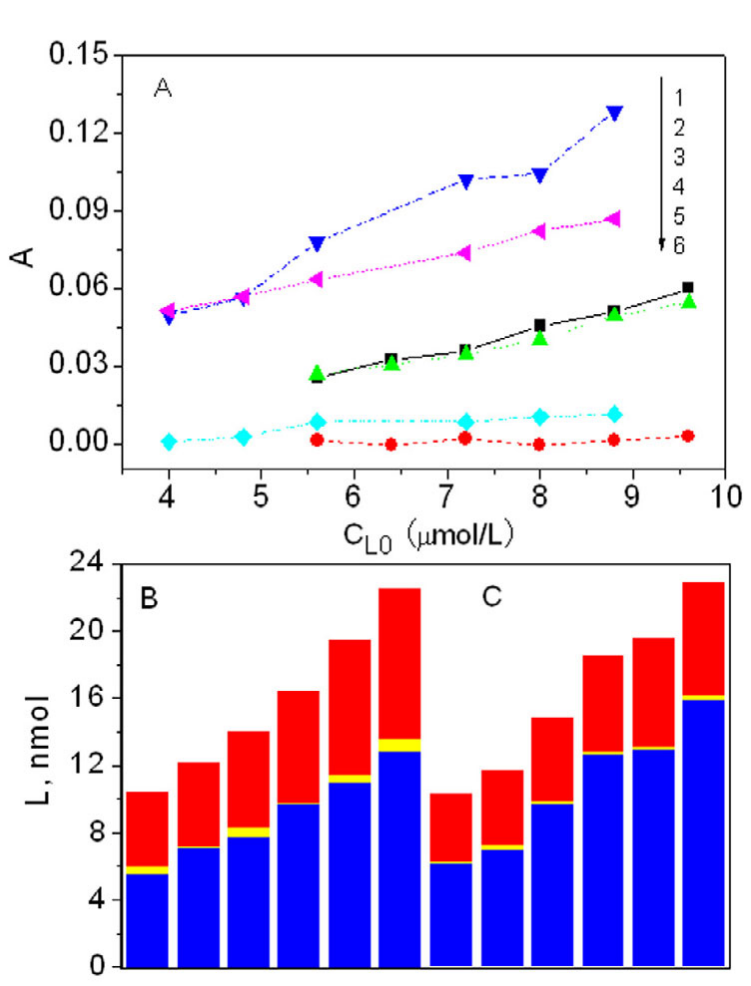
**Spectrophotometric measurements of the solutions containing *E. coli *and Sudan red compounds**. 1: absorption of Sudan IV in extracellular solution; 2: absorption of Sudan IV accumulated in membrane; 3: absorption of Sudan II free in extracellular aqueous medium; 4: absorption of Sudan II accumulated in membrane; 5: absorption of Sudan IV in cytosol; 6: absorption of Sudan II in cytosol. B: distribution of Sudan II in the extracellular solution (red), membrane bilayer (blue) and cytosol (yellow). C: the equivalent information as given in B, but for Sudan IV.

The toxicant in the extracellular medium enters the cells when possible by passive diffusion [23] and accumulates until the cell dies. In this work, the mixture of Sudan red compounds with *E. coli *was sampled at 30 min of culture time and treated according to standard procedures. Over 98% of the *E. coli *in suspensions (1) and (4) was pelleted by centrifugation at 3500 × g for 5 min, as determined by comparing the absorptions before and after centrifugation. Similarly, over 98% of the ultrasonication fragments in suspension (8) could be separated by centrifugation for 5 min at 5600 × g. After fractionation, the absorptions of the Sudan red compounds in the extracellular solution, membrane and cytosol were measured as shown in Figure 6(A). Using Eqs. 2 – 5 in Table 1, the distributions of the Sudan red compounds were calculated as shown in Figure 6(B & C). Over 60% of the Sudan II in the medium penetrated into the cell; 90% of this remained in the membrane. Similarly, from the columns in C, more than 75% of the Sudan IV entered the cells and about 95% remained in the membrane. This results from the Langmuir adsorption of Sudan red compounds on to the membrane phospholipids via non-covalent interactions as described above. The binding model is sketched in Figure 3(3). As a result, only a small amount of Sudan red compounds enters the *E. coli *cytosol, as shown by the small yellow parts of Figure 6(B and C), i.e. entry into the cytosol may be impeded for Sudan red compounds. While it is possible for such compounds to cause membrane toxicity, it is difficult to identify the target biomacromolecular toxicity, *e.g*. DNA damage, directly.

### Interaction of Sudan red compounds with cells

**Figure 7:**
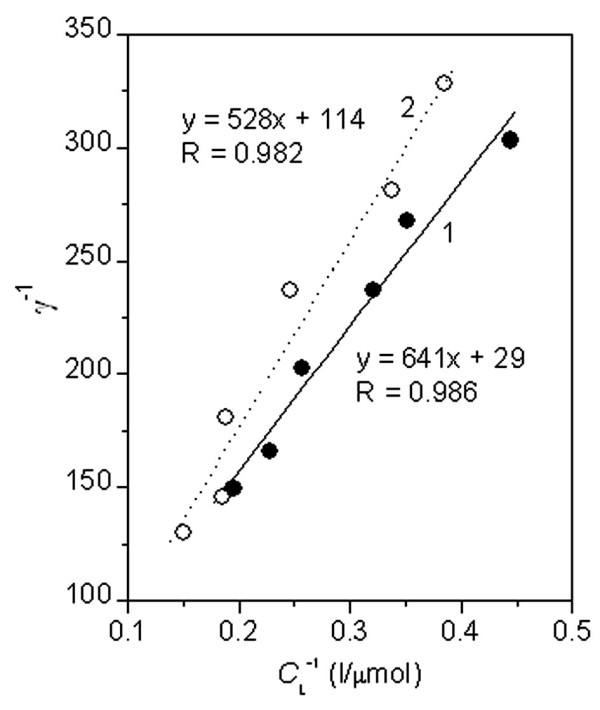
**Linear regression curves for the interactions of Sudan red compounds with *E. coli***: A: Sudan II; B: Sudan IV.

In order to investigate the type of interaction between the Sudan red compounds and the membrane, the membrane phospholipids must be identified. The membrane was isolated according to standard procedures and the final preparation was dissolved in dichloromethane. The molar quantity of phosphorus in the membrane was determined by inductively coupled plasma – optical emission spectroscopy (ICP-OES) [24]. The results showed that 5 ml of *E. coli *culture medium contained 1.32 μmol of membrane phosphorus. From this, the values of *γ *in the Sudan compound-*E. coli *mixtures were calculated, and plots of *γ*^-1 ^vs. *C*_L_^-1 ^are shown in Figure 7. Obviously, the aggregation of Sudans II and IV in the cell membranes obeyed the Langmuir adsorption isotherm, as in the liposome preparations. The binding constants were calculated to be *N*_Sudan-II _= 1/29, *N*_Sudan-IV _= 1/114 and *K*_Sudan-II _= 1.86 × 10^4^, *K*_Sudan-IV _= 6.02 × 10^4^. The binding of Sudan IV in the cell membrane is more stable than that of Sudan II but a greater molar amount of Sudan II is bound. Comparison with Figure 2 shows that the *N *and *K *values of Sudan II in the cell membrane are very similar to those in the lecithin liposomes. For Sudan IV, however, *N *is higher and *K *is lower in the living cell membrane than in the lecithin liposomes. The membrane proteins may decrease the steric effect of the long Sudan IV molecules and increase the area covered. In addition, cell movement will continuously alter the surface-binding position and pattern of the long Sudan IV molecules in order to accommodate the adsorption of more of these molecules.

## Conclusion

Penetration into the cytosol is impeded for Sudan red compounds, and it is possible for them not to cause the terminal toxicity. This work provides a useful experimental strategy for the quantitative assessment of potential membrane toxicity of hydrophobic compounds with polar groups and recognition of the terminally toxic substances. This technique may be useful in a variety of research fields including environmental and life sciences, and medicine.

## Methods

### Materials and instruments

Lecithin (Sinopharm Cheam), 20 mg/ml, was suspended in deionized water and then dispersed ultrasonically at maximal amplitude at 4°C for 5 cycles of 15 s interspersed with 45 s periods of rest [12]. Stock solutions of Sudan II (0.80 mM) and Sudan IV (0.10 mM) (Shanghai Chemical Reagents Center of National Medicine Group of China) were prepared in absolute ethanol. Britton-Robinson (B-R) buffers at pH 2.18, 3.13, 4.16, 5.72, 7.16, 8.69, 9.62 and 10.38 were prepared and were used to examine the effect of solution pH on the interactions of the Sudan red compounds with liposomes. A pH 7.0 PBS was prepared by mixing 0.8% NaCl, 0.02% KCl, 0.0144% Na_2_HPO_4 _and 0.024% KH_2_PO_4 _and was used to re-suspend bacterial pellets. Sterile L-B nutrient medium containing 1.0% peptone, 0.5% yeast extract and 1.0% NaCl was prepared and used for culturing *E. coli*. The *E. coli *strain used was provided by the Environmental Biological Laboratory of Tongji University.

A Model Lambda-25 spectrometer (Perkin-Elmer Instruments, USA) with version-2.85.04 UV-WinLab software was used to record the absorption spectra of the Sudan solutions and to measure turbidities and absorptions. A Model JY92-II Ultrasonic Cell Disruptor (Ningbo Xinzhi Instruments, China) was used to disrupt the bacterial cells. A Model Anke TGL-16C High-speed Centrifuge (Shanghai Anting Sci. Instruments, China) with 7.0 ml plastic tubes was used to separate the liposomes and cell membranes. Solution pHs were measured with a Model pHS-25 Acidity Meter (Shanghai Precise Instruments., China). A Model TS-030 Thermostat Cycling Bath (Shanghai Yiheng S&T, China) was used to maintain the experimental temperature between room temperature and 70°C. A Model SK3300H Ultrasonic Cleaning Device (Shanghai Ultrasonic Cleaning Instruments, China) was used to accelerate the dissolution of solutes and to disperse the phospholipids. A Model CHA-2 Thermostat Gas Bath Vibrator (Jintan E-tong Electrons, Jiangsu, China) and a Model SPX-80BS-II Biochemical Incubator (Shanghai Cimo Medical instruments, China) were used to culture the bacteria. A Model Optima 2100 DV ICP- OES (PerkinElmer Instruments, USA) was used to measure the phosphorus content of the membrane phospholipids. A Model GM-100 diaphragm vacuum pump (Tengda Filtration Devices of Tianjin, China) was used to volatilize the organic reagent in the membrane extracts to yield the *E. coli *membrane residue.

### In-vitro interaction of Sudan red compounds with liposomes

A series of mixtures containing 50 μl of liposome suspension and aliquots of 0.8 mM Sudan II (0 to 120 μl) were diluted to 5.00 ml with deionized water. After mixing for 10 s in the ultrasonic cleaning device, they were centrifuged for 20 min at 7000 × *g*. Each pellet was dissolved in 5.00 ml of ethanol. A dual-wavelength spectral correction method [18] was used to eliminate background interference. The solutions were measured at 400 and 495 nm, and a standard curve of Sudan II in the liposomes was generated. Applying the same procedures, a standard curve prepared from aliquots of the 0.1 mM stock solution of Sudan IV was used to measure the interaction of Sudan IV with the liposomes, except that data were collected at 400 and 515 nm.

### Culture of *E. coli *and determination of membrane phospholipids

*E. coli *was selected as the model microorganism for further experiments with the Sudan dyes. Several cycles of the *E. coli *strain were grown in 250 ml of L-B nutrient medium in culture bottles in the thermostated gas bath vibrator at 37°C. Bacterial growth was monitored by turbidimetric measurement at 600 nm. The *E. coli *cultures were also treated with MTT [22] and the absorbance measured at 555 nm to determine the number of living cells.

After the *E. coli *growth reached stationary phase, 5.00 ml of the zymotic fluid from the culture bottle was placed in a 7-ml centrifuge tube. The sample was centrifuged for 5 min in 3500 × g to sediment the bacteria, and the yellow supernatant was removed. The pellet was resuspended with PBS (2.5 ml), mixed well, and centrifuged again to obtain a pellet. The pellet was suspended and dispersed in PBS (2.5 ml) by ultrasonication for 10 × 5 s at 200 w interspersed by 10 s intervals of rest. After centrifugation, the supernatant was removed, the pellet was dissolved in 2.50 ml dichloromethane and the organic phase was volatilized under vacuum. The dried residue was treated [25] with 4 ml H_2_SO_4_, 4 ml HCl and 5 ml water to prepare a clear solution. The concentration of phosphorus was determined by ICP-OES [24]. From this, the molarity of the membrane phospholipid species in the *E. coli *culture medium was calculated.

### Interactions of Sudan red compounds with *E. coli*

**Figure 8:**
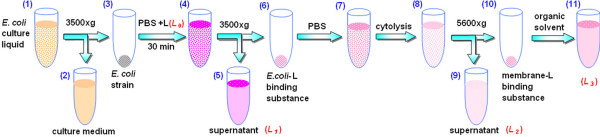
**Cartoon illustrating the separation and detection procedures used to investigate the interaction between *E. coli *and Sudan red compounds (L)**. (1) *E. coli *culture medium; (2) supernatant of (1) after centrifugation at 3500 × g; (3) *E. coli *collected; (4) *E. coli *liquid re-suspended with PBS still containing Sudan dyes (L) ; (5) supernatant of (4) after centrifugation at 3500 × g; (6) *E. coli *– L binding substance collected; (7) *E. coli *– L binding liquid re-suspended with PBS; (8) *E. coli *– L binding liquid after ultrasonication; (9) supernatant of (8) after centrifugation at 5600 × g; (10) membrane – L binding substance collected and (11) membrane – L binding solution dissolved in organic solvent.

Five ml of the zymotic fluid was centrifuged for 5 min at 3500 × g (Figure 8) after *E. coli *growth reached stationary phase. The yellow supernatant was removed and the *E. coli *pellet (3) was re-suspended in 2.5 ml of PBS. Aliquots (0–120 μl (*L*_0_)) of Sudan II (L) were added. After incubation in an isothermal shaker bed at 37°C for 30 min, the liquid was centrifuged for 5 min at 5600 × g. The absorbance of the supernatant (5) was measured at 495 nm. The amount (*L*_1_) of free Sudan II was calculated by Eq. 2 as shown in Table 1. The pellet (6) was again re-suspended in 2.5 ml of PBS (7) and centrifuged as above, and the clear solution was removed. PBS (2.5 ml) was added to the centrifuge tube and the cells were ruptured by ultrasonication for 5 × 5 s at 200 w interspersed with 10 s rest periods. The liquid (8) was centrifuged for 5 min at 5600 × g. The absorbance of the supernatant (9) was measured according to the above method and the amount (*L*_2_) of Sudan II in the cytosol calculated using Eq. 3. The pellet (10) was dissolved in 2.50 ml of ethanol to prepare a clear solution. The amount (*L*_3_) of Sudan II accumulated in the membrane was calculated by Eq. 2 from the absorbance of this solution (11). Simultaneously, Sudan IV and a blank without L were processed by the same procedures, except that Eqs. 5 and 4 were used instead of Eqs. 3 and 2 to calculate the amounts of Sudan IV in the intracellular liquid, cytosol and membrane. Thus, the distributions of Sudan II and IV were determined.

## Authors' contributions

LL performed all of the experimental operations and treated the data. HWG designed the experimental procedures, created the cartoon illustration and performed writing of the manuscript. JRR performed the preparation of lecithin liposome liquid and optimization of the centrifugation condition. LC collected and analyzed the data. YCL provided the financial support for this work. JFZ assisted the English editing and polishing of this manuscript. HPZ performed the culture of *E. coli *strain. YY provided the research tools for this work. All authors read and approved the final manuscript.

## Figures and Tables

**Table 1:** Standard curves for determination of Sudan red compounds in ethanol and pH 7.0 phosphate buffer solution (PBS)

Chemical	Upper limit, nM	Wave- length, nm	Medium	Linear equations	Linear correlation coefficient
Sudan II	40	495	ethanol	*A *= 0.0155*C*_L _(2)	0.9982
Sudan II	1.2	495	PBS *	*A *= 0.0121*C*_L _- 0.001 (3)	0.9993
Sudan IV	0.6	515	ethanol	*A *= 0.0318*C*_L _- 0.0018 (4)	0.9990
Sudan IV	5	515	PBS	*A *= 0.0196*C*_L_- 0.0007 (5)	0.9990
